# Pediatric Mild Traumatic Brain Injury and Concussion: Modern Pathophysiological Insights, Diagnostic Advances, and Active Management Protocols

**DOI:** 10.3390/brainsci16090998

**Published:** 2026-09-21

**Authors:** Mirjana Raicevic, Srdjan S. Nikolovski

**Affiliations:** 1 Department of Neurosurgery, University Children’s Hospital, Tirsova St. 10, 11000 Belgrade, Serbia; neuromira@gmail.com; 2 Faculty of Medicine, University of Belgrade, Dr. Subotica St. 8, 11000 Belgrade, Serbia; 3 Department of Pathology and Laboratory Medicine, Loyola University Chicago Medical Center, 2160 S First Ave, Maywood, IL 60153, USA; 4 Serbian Resuscitation Council, Dr. Djordja Joanovica St. 2, 21000 Novi Sad, Serbia; 5 Faculty of Pharmacy Belgrade, University Educons, Poenkareova St. 14, 11000 Belgrade, Serbia

**Keywords:** pediatric concussion, mild traumatic brain injury, neurometabolic cascade, Child SCOAT6, return-to-learn, return-to-sport, blood biomarkers, sub-threshold exercise

## Abstract

**Highlights:**

**What are the main findings?**

- Pediatric concussion pathophysiology involves a neurometabolic cascade (ionic imbalance, mitochondrial dysfunction, diffuse axonal injury, neuroinflammation) magnified by unique neurodevelopmental vulnerabilities.
- Management has evolved from prolonged rest to early active recovery, incorporating brief rest (24–48 h), sub-symptom aerobic exercise, return-to-learn accommodations, and stepped return-to-sport protocols.

**What are the implications of the main findings?**

- Clinical evaluation should utilize age-appropriate tools (Child SCAT6/Child SCOAT6), clinical domain profiling, and emerging blood biomarkers (GFAP, UCH-L1, NfL), while neuroimaging should be guided by validated decision rules.
- Individualized rehabilitation and early risk stratification can optimize recovery and prevent cumulative injury, supporting the need for standardized pediatric biomarker thresholds and prospective validation studies.

**Abstract:**

Traumatic brain injury and pediatric concussion represent major global public health concerns. This narrative review aims to provide an updated overview of pediatric concussion by integrating current evidence regarding pathophysiology, diagnostic advances, neuroimaging, biomarkers, and evidence-based management strategies. Concussion pathophysiology involves a complex neurometabolic cascade characterized by ionic imbalance, mitochondrial dysfunction, cerebral blood flow alterations, diffuse axonal injury, and neuroinflammation. In pediatric populations, unique neurodevelopmental vulnerabilities (higher head-to-torso ratio, weaker cervical musculature, ongoing myelination) magnify these disturbances. Contemporary management has evolved from prolonged rest to early active recovery, incorporating brief initial rest (24–48 h) followed by sub-symptom threshold aerobic exercise, structured return-to-learn accommodations, and stepped return-to-sport protocols. Emerging diagnostic tools include the Child SCAT6/Child SCOAT6, vestibular/ocular motor screening, and blood biomarkers (GFAP, UCH-L1, NfL). Future research should focus on standardizing pediatric biomarker thresholds and validating multimodal diagnostic algorithms through prospective multicenter studies.

## 1. Introduction

Traumatic brain injury (TBI) represents a major global health burden, with contemporary epidemiological estimates indicating over 20 million incident TBI cases annually worldwide, with sports-related concussion (SRC) accounting for a substantial proportion among children and adolescents [1,2].

There is currently a large pool of evidence demonstrating the mechanisms underlying mild TBI (mTBI) and concussion, as well as their long-term consequences [3,4,5]. However, the pathophysiology still remains under investigation, although it is certain that biomechanical forces cause changes starting at the level of the individual cell, affecting metabolism and neuronal vitality.

While clinical recovery typically occurs within 2 to 4 weeks, the management paradigm has fundamentally shifted away from historical prolonged passive rest toward early, targeted active recovery. In this review, integration of the 6th International Consensus Statement on Concussion in Sport (Amsterdam 2022/2023) framework is operationalized across four key clinical pillars: (1) transitioning from rigid rest to brief relative rest (24–48 h) followed by early sub-symptom threshold aerobic exercise; (2) retiring obsolete numerical severity grading in favor of multidomain clinical symptom profiling (somatic, cognitive, vestibular-ocular, affective, sleep-related); (3) deploying age-tailored diagnostic toolsets (Child Sport Concussion Assessment Tool 6 [SCAT6] and Child Sport Concussion Office Assessment Tool 6 [SCOAT6]) across acute and subacute clinical settings; and (4) enforcing structured, step-wise return-to-learn (RTL) progression as an obligatory prerequisite prior to return-to-sport (RTS) clearance [6,7,8,9]. Throughout this manuscript, these consensus pillars are systematically linked to pediatric-specific neurodevelopmental vulnerabilities, blood biomarker kinetics, and individualized rehabilitation protocols.

Despite substantial advances in concussion research over the past decade, several critical scientific and clinical gaps hinder optimal pediatric care:

- Scientific Gaps: The literature remains fragmented, leaving a significant disconnect between basic neurometabolic cascade mechanisms (e.g., acute energy crises, microglial activation, and microstructural white matter degradation) and clinical symptom domain profiles [10,11,12]. Furthermore, while blood-based biomarkers (such as glial fibrillary acidic protein [GFAP], ubiquitin carboxy-terminal hydrolase L1 [UCH-L1], and neurofilament light chain [NfL]) show high diagnostic promise, pediatric-specific baseline reference intervals, age-dependent clearance kinetics, and validated diagnostic cut-offs remain incompletely defined relative to adult standards [13,14,15].
- Clinical Gaps: Translating updated international guidelines into routine pediatric practice remains inconsistent. Clinicians routinely encounter difficulties operationalizing the paradigm shift from historical strict rest to early sub-symptom threshold exercise, integrating validated imaging decision rules (e.g., PECARN) alongside novel fluid biomarkers, and implementing newly standardized diagnostic instruments (Child SCAT6 and Child SCOAT6) in acute and subacute settings [6,8,9,16,17].

Furthermore, practical guidelines outlining how to weigh and sequence diagnostic tools (PECARN, Child SCOAT6, Vestibular/Ocular Motor Screening [VOMS], fluid biomarkers) within an integrated clinical workflow remain lacking.

To address these limitations, the novelty and primary contribution of this review lie in providing a unified, age-tailored synthesis that directly links developmental pathophysiological mechanisms to actionable clinical management. By integrating the 6th International Consensus Statement on Concussion in Sport (Amsterdam 2022/2023) framework, age-stratified biomarker profiles, specialized pediatric diagnostic toolsets, and step-wise RTL and RTS algorithms [1,7], this article offers an all-in-one diagnostic and therapeutic roadmap designed specifically for pediatric practitioners and clinical researchers.

Specifically, the intended scope of this narrative review encompasses pediatric mTBI and concussion broadly, across all mechanism etiologies (including falls, motor vehicle collisions, and recreational injuries). However, because SRC represents the primary model and major evidence base driving international consensus frameworks (e.g., Amsterdam 2022/2023) and active rehabilitation protocols, findings from SRC studies are prominently integrated while highlighting their clinical applicability across the entire pediatric mTBI spectrum.

## 2. Literature Search Strategy

To ensure a comprehensive, rigorous, and reproducible synthesis of the literature, this narrative review was structured in alignment with the Scale for the Assessment of Narrative Review Articles guidelines [18].

**Database Coverage & Search Execution:** A systematic search was performed across PubMed/MEDLINE, Scopus, Web of Science, and Google Scholar for articles published between 1 January 2011 and 31 December 2025, with a final search update conducted on 15 January 2026.

**Search Terms & Boolean Logic:** A combination of Medical Subject Headings and free-text keywords was operationalized using Boolean operators (AND, OR):

- *Population:* (“pediatric” OR “child” OR “adolescent” OR “youth” OR “children”);
- *Condition:* (“mild traumatic brain injury” OR “mTBI” OR “concussion” OR “sports-related concussion” OR “SRC”);
- *Pathophysiology:* (“pathophysiology” OR “neurometabolic cascade” OR “diffuse axonal injury” OR “microstructural” OR “neuroinflammation” OR “blood-brain barrier” OR “excitotoxicity”);
- *Diagnostics and Biomarkers:* (“neuroimaging” OR “diffusion tensor imaging” OR “DTI” OR “PECARN” OR “biomarkers” OR “GFAP” OR “UCH-L1” OR “neurofilament light” OR “NfL” OR “S100B” OR “Child SCAT6” OR “Child SCOAT6”);
- *Management and Intervention:* (“management” OR “active recovery” OR “sub-symptom threshold exercise” OR “return-to-learn” OR “return-to-sport” OR “rehabilitation”).

GFAP and UCH-L1 were explicitly included alongside general biomarker search terms due to their status as the primary FDA-cleared fluid biomarkers for acute intracranial injury triage, offering complementary cellular specificity (astrocytic structural damage versus acute neuronal injury) and distinct temporal release kinetics.

**Inclusion & Exclusion Criteria:**

- *Inclusion Criteria:* (1) Peer-reviewed human studies focusing on pediatric or adolescent populations (aged 0–18 years) with mTBI or concussion; (2) Systematic reviews, meta-analyses, international consensus statements, clinical practice guidelines, randomized controlled trials, and high-quality prospective/retrospective cohort studies; (3) Articles published in English; (4) Landmark foundational studies establishing essential pathophysiological mechanisms, regardless of publication date.
- *Exclusion Criteria:* (1) Studies focusing exclusively on moderate-to-severe TBI or penetrating head trauma without disaggregated mTBI data; (2) Preclinical animal models unless required to explain cellular/neurometabolic mechanisms where human data were lacking; (3) Non-peer-reviewed literature, conference abstracts without full text, editorials, and non-English publications.

**Study Selection & Quality Appraisal:** Two authors (M.R. and S.S.N.) independently screened titles and abstracts retrieved from the initial search queries. Full-text articles were subsequently evaluated against the predefined eligibility criteria. Discrepancies regarding article inclusion were resolved through mutual consensus and critical re-examination of the full text. Additionally, reference lists of key consensus statements and systematic reviews were manually cross-referenced to retrieve additional relevant studies.

## 3. Definition of Concussion and Traumatic Brain Injury

TBI is defined as an alteration in brain function, or other evidence of brain pathology, caused by an external force [7]. This encompasses a spectrum of injuries ranging from mild to severe. The most widely accepted classification system utilizes the Glasgow Coma Scale (GCS) [19], with mTBI defined by a GCS score of 13–15, moderate TBI by a GCS of 9–12, and severe TBI by a GCS of 3–8 [20]. While moderate and severe TBI are typically associated with macroscopic structural abnormalities on conventional neuroimaging, mTBI (and concussion specifically) is characterized by functional and microstructural disturbances without gross anatomical lesions [7,10].

Within the broad spectrum of pediatric mTBI, concussion represents a distinct functional subcategory caused by transmitted biomechanical forces resulting from direct or indirect head impacts [7,10]. In SRC, the most extensively evaluated subset of pediatric mTBI, the 6th International Consensus Statement (Amsterdam 2022/2023) defines injury as an impulsive force transmitted to the head, neck, or body resulting in transient functional neurometabolic and microstructural disturbances rather than macrostructural gross anatomical lesions [7,10]. Historical rigid numerical grading systems (e.g., Cantu, American Academy of Neurology) and same-day RTS criteria are now clinically obsolete and explicitly prohibited in pediatric populations [1,7].

Instead of numerical severity grading, modern concussion classification relies on clinical domain profiling, categorizing injury manifestation into distinct domains to guide targeted multidisciplinary intervention [7,8].

Contemporary understanding recognizes concussion as a dynamic pathophysiological process rather than a static injury classification. This conceptual shift has important implications for individualized assessment, prognosis, and treatment, replacing historical numerical grading systems with symptom-domain profiling and targeted multidisciplinary rehabilitation. Rather than attempting to classify injury severity using a single variable such as loss of consciousness (LOC) or symptom duration, current clinical practice integrates multiple functional domains to guide personalized management strategies. The updated clinical domain profiling in pediatric concussion is presented in Table 1.

In clinical practice and the current literature, concussion and mTBI are frequently used interchangeably; however, contemporary consensus guidelines define concussion as a functional clinical subset within the broader mTBI spectrum [1,7,21]. Mild TBI is the umbrella clinical classification defined by objective initial post-injury parameters (GCS score 13–15, LOC < 30 min, post-traumatic amnesia < 24 h) [20,21]. Concussion represents a pathophysiological subcategory of mTBI caused by transmitted biomechanical forces, characterized primarily by acute, transient neurometabolic, and microstructural disturbances in the absence of macroscopic structural lesions on routine CT or MRI [7,10]. Crucially, under the Amsterdam 2022/2023 framework, concussion and mTBI are not differentiated on the basis of symptom duration or recovery trajectory, as persistent post-concussive symptoms (PPCSs) can occur across both constructs regardless of initial clinical subset classification [7,22].

Overall, modern definitions conceptualize traumatic brain injury across a continuous clinical and pathophysiological spectrum, ranging from gross macrostructural lesions in moderate-to-severe TBI to transient, predominantly functional microstructural and neurometabolic disturbances characteristic of mild TBI. Within this broader continuum, concussion is defined as a specific functional subset of mild TBI driven by transmitted biomechanical forces. Recognizing this diagnostic hierarchy and dynamic pathophysiological continuum has shifted modern clinical practice away from rigid numerical severity grading toward individualized, domain-based risk assessment and functional recovery tracking.

## 4. Pathophysiology and Neurometabolic Basis of Cerebral Concussion

The pathophysiological cascade following pediatric concussion is initiated by biomechanical forces that trigger immediate, acute cellular and neurometabolic alterations [10,23,24,25,26]. Current understanding of these mechanisms (including ionic fluxes, excitotoxicity, acute metabolic shifts, and cerebral blood flow [CBF] dysregulation) is largely derived from experimental animal models and adult clinical cohorts [10,23,24,25,26]. While these fundamental cellular responses are believed to be broadly conserved across the lifespan, caution is warranted when translating preclinical and adult timeline data directly to pediatric patients, in whom direct in vivo human evidence remains more limited [10,27].

**Figure 1:**
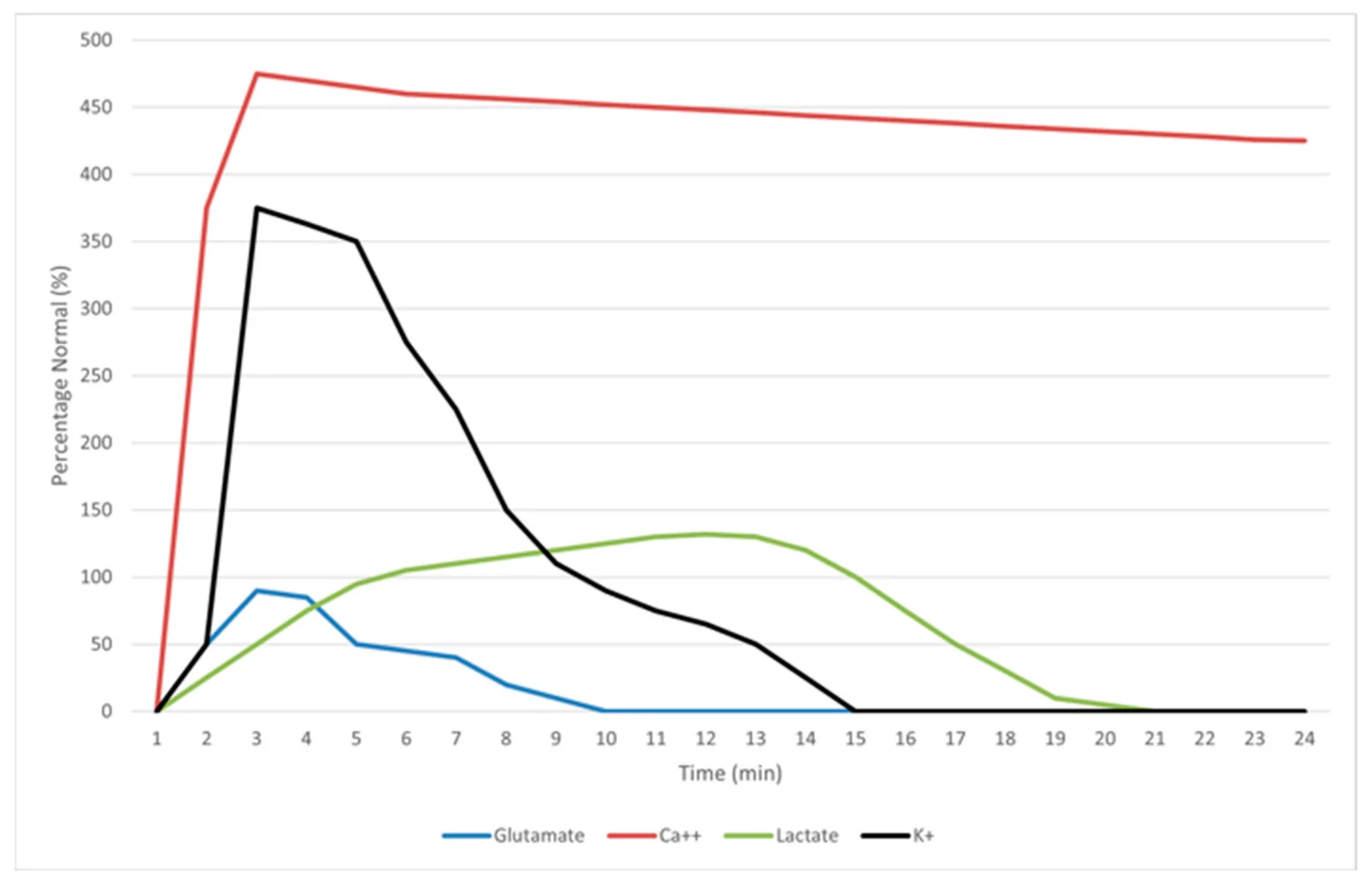
Conceptual schematic model of hyperacute neurometabolic kinetics and temporal phase transition during the immediate post-concussive window (0–24 min). Note: This figure is a normalized conceptual schematic designed for illustrative visualization; plotted curves do not represent direct quantitative human pediatric measurements or unadjusted raw clinical experimental data. The *y*-axis (“Percentage Normal (%)”) represents normalized relative percentage changes relative to pre-injury baseline (100%). Data points were plotted to schematically map established temporal kinetic trends reported in rodent fluid-percussion injury and cortical microdialysis models [10,23,24,26]. Biomechanical impact triggers rapid depolarization, driving hyperacute extracellular potassium (K^+^) efflux (peaking within 1–2 min), indiscriminate glutamate release, and intracellular calcium (Ca^++^) influx. This ionic disruption forces rapid lactate accumulation and transient hyperglycolysis to meet surging Na^+^/K^+^-ATPase pump demands, directly initiating cellular energy mismatch. Data derived conceptually and digitized from Katayama et al. [23], Kawamata et al. [24], Hovda et al. [26], and Giza & Hovda [10].

The neurometabolic cascade described above provides biological explanations for common clinical presentations. The hyperacute phase (0–24 min post-injury, Figure 1) is dominated by rapid ionic shifts (K^+^, Ca^++^), massive glutamate release, and early lactate accumulation that trigger acute hyperglycolysis [10,23]. Following this initial cellular depolarization, a secondary phase of prolonged cerebral hypometabolism ensues (typically peaking between days 3 and 10), which coincides with the period of greatest symptom burden, cognitive dysfunction, and vulnerability to secondary injury [7,10]. The vulnerability period following the initial injury, when cells are more susceptible to a second insult, provides the biological rationale for the strict avoidance of same-day RTS and graduated return-to-activity protocols [7,10]. The relationship between diffuse axonal injury (DAI) and persistent cognitive symptoms, particularly processing speed and executive function deficits, reflects the disruption of white matter tracts connecting prefrontal and subcortical regions. Diffusion tensor imaging (DTI) studies have demonstrated that children with concussion exhibit microstructural abnormalities in thalamo-prefrontal tracts that are associated with working memory deficits. Furthermore, DTI metrics have been shown to correlate with processing speed and executive function performance in pediatric TBI populations, highlighting the clinical significance of white matter microstructural integrity for cognitive recovery following concussion [12]. These dynamic shifts are schematically illustrated in Figure 1 as a normalized conceptual model (where parameters depict relative directional changes from a 100% pre-injury baseline across 0–24 min). Rather than raw human data, the plotted curves represent digitized, normalized schematic values derived from published kinetic trends in preclinical fluid-percussion injury and cerebral microdialysis models [10,23,24,26], tracking the hyperacute spike of extracellular potassium (K^+^), indiscriminate glutamate release, intracellular calcium (Ca^++^) influx, and early lactate accumulation.

**Figure 2:**
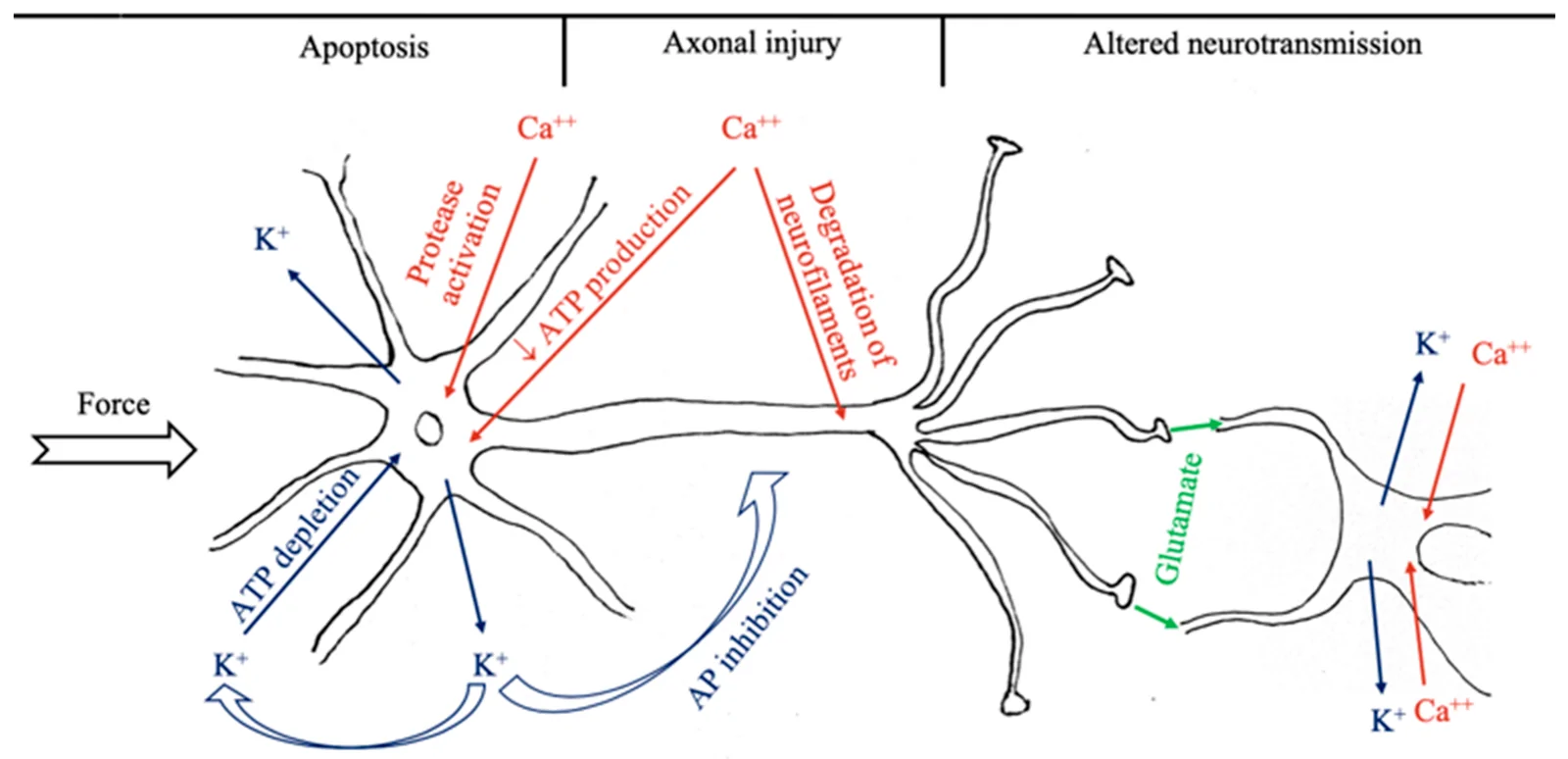
Mechanistic model of the acute cellular energy crisis following concussion. Mechanical membrane shear leads to massive ionic shifts and uncoupled neurotransmitter release. The resulting surge in Na^+^/K^+^-ATPase activity drastically increases ATP demand. Concurrently, impaired mitochondrial oxidative phosphorylation forces reliance on transient anaerobic glycolysis, causing lactate accumulation and tissue acidosis. This metabolic supply–demand mismatch creates an acute window of cellular vulnerability to secondary insults. Legend: ATP—adenosine-triphosphate, AP—action potential.

**Figure 3:**
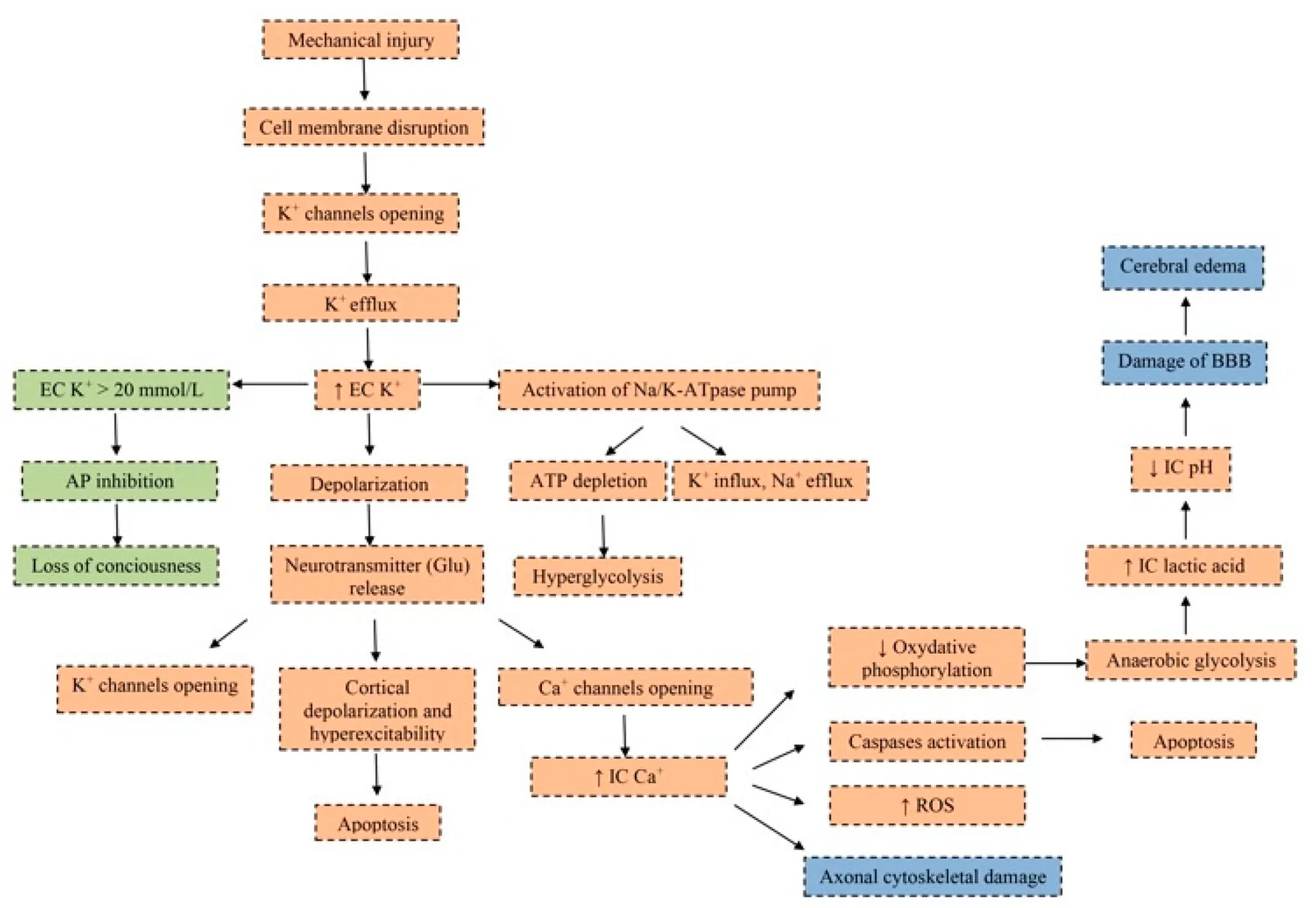
Hierarchical multi-level model of the post-concussive cascade. The schematic illustrates functional progression across three visually distinct biological and clinical tiers highlighted by background shading and color coding: Level 1 (Cellular and Molecular, orange): Hyperacute ionic fluxes, excitotoxicity, mitochondrial dysfunction, and reactive oxygen species generation; Level 2 (Microstructural and Tissue, blue): Neuroinflammation, blood–brain barrier disruption, and diffuse axonal injury; and Level 3 (Clinical and Biomarker Domain, green): Systemic biofluid biomarker release (GFAP, UCH-L1, NfL) and multi-domain clinical symptom expression. Legend: EC—extracellular, IC—intracellular, ATP—adenosine-triphosphate, AP—action potential, Glu—glutamate, ROS—reactive oxygen species, BBB—blood–brain barrier.

It is important to emphasize that while the underlying cellular framework (Figure 1, Figure 2 and Figure 3) provides a valuable conceptual baseline, the precise temporal kinetics, magnitude, and recovery trajectory of this neurometabolic crisis in children are distinctly modulated by ongoing neurodevelopment [10,27]. Features unique to the maturing brain (such as higher baseline glucose metabolic demands, incomplete myelination, and evolving receptor subunit profiles) mean that pediatric neural tissue may exhibit heightened sensitivity or altered temporal dynamics compared to adult or animal models [1,10].

As illustrated across Figure 1, Figure 2 and Figure 3, the post-concussive cascade is organized across distinct biological and clinical levels that evolve through temporally linked phases. The hyperacute ionic flux and indiscriminate glutamate release (Figure 1) directly drive an acute cellular energy crisis (Figure 2). As detailed in Figure 3, these events translate across three hierarchical levels: initial cellular and molecular disruptions (Level 1) induce secondary tissue-level neuroinflammation, blood–brain barrier permeability, and microstructural axonal strain (Level 2), which ultimately propagate into systemic biofluid biomarker release and multi-domain clinical symptom profiles (Level 3).

In the pediatric population, this acute energy crisis is particularly magnified due to unique neurodevelopmental and biomechanical vulnerabilities. Children possess higher head-to-torso mass ratios, weaker cervical musculature, incomplete cranial vault ossification, and ongoing myelination [1,10]. Lower baseline axonal myelination renders unmyelinated or sparsely myelinated pediatric nerve fibers far more susceptible to mechanical shear stress and axolemmal ionic leakage. Furthermore, because the developing brain has higher baseline glucose and oxygen metabolic demands to support active synaptogenesis, axonal myelination, and dendritic arborization, any post-injury metabolic mismatch (elevated glycolysis coupled with reduced CBF) induces a prolonged energy deficit, explaining the extended recovery windows frequently observed in young athletes [7,10,27,28].

In the acute period after impact (3–5 min), mechanical injury disrupts the cell membrane, causing the opening of voltage-gated potassium channels, which results in massive potassium efflux, increased extracellular potassium levels, neuronal depolarization, and neurotransmitter release [10,23,26,29]. In order to correct steady-state potential, the sodium/potassium adenosine-triphosphatase pump is activated, causing an influx of potassium back in the cell in exchange for intracellular sodium (Figure 1, Figure 2 and Figure 3). This process requires large amounts of adenosine triphosphate (ATP), which neurons produce mainly through the process of transient hyperglycolysis during the first 30 min to 4 h after injury and can be detected by a positron-emission tomography scan. Inhibition of the action potential and LOC may occur when the extracellular potassium concentration accumulates above 4–5 mmol/L to levels greater than 20 mmol/L, which, in certain situations, may take only several seconds [30].

Initial depolarization leads to massive excitatory neurotransmitter release (mainly glutamate) resulting in excitotoxicity, and possibly permanent damage [29]. Glutamate induces ligand-gated potassium channel opening and further potassium efflux, but also binds to N-methyl-D-aspartate receptors, allowing for unrestricted cortical depolarization and hyperexcitability, which leads to cell damage and apoptosis (Figure 1, Figure 2 and Figure 3) [26]. This process of altered neurotransmission is detectable by functional magnetic resonance imaging (MRI).

Increased release of excitatory neurotransmitters also results in the accumulation of intracellular calcium, leading to several consequences. It triggers a cascade of secondary metabolic disruptions. Mitochondrial calcium overload inhibits oxidative phosphorylation, impairing ATP production and driving anaerobic glycolysis with resultant lactate accumulation and tissue acidosis [31]. Concurrently, elevated cytosolic calcium drives the generation of reactive oxygen species and activates calcium-dependent proteases, including calpains and caspases, which induce apoptosis (Figure 2) [31,32]. These proteolytic cascades degrade axonal neurofilaments and microtubular structures, leading to progressive axolemmal swelling, focal transport failure, and eventual secondary axotomy, a microstructural degradation detectable in vivo via DTI [33].

In the first four days after brain injury, intracellular magnesium concentration also decreases (identifiable by magnetic resonance spectroscopy), resulting in a decreased intensity of glycolysis and oxidative phosphorylation, and eventually in an even more severe cellular energy crisis [10,34,35].

An accompanying decrease in CBF results in lactate accumulation and intracellular acidosis [36,37]. After this initial phase, a state of cerebral hypometabolism occurs and may last up to 10 days [24]. This phase is characterized by decreased protein synthesis and reduced oxidative capacity. As a consequence, cells are still viable, but more susceptible to death after a second (even sub-concussive) insult [26].

Collectively, these early metabolic disturbances initiate a complex cascade involving ionic imbalance, mitochondrial dysfunction, impaired cerebral perfusion, oxidative stress, and excitotoxicity. While these hyperacute cellular perturbations occur across all traumatic brain injuries, DAI and the acute neuroinflammatory response are specifically highlighted in dedicated subsections below due to their pivotal role as translational bridges linking acute cellular distress to clinical outcomes and diagnostic modalities.

1. DAI represents the primary microstructural substrate connecting biomechanical shear strain to white matter tract disruption. DAI directly explains domain-specific cognitive deficits (such as reduced processing speed and working memory) and provides the biological rationale for advanced neuroimaging (DTI) and axonal fluid biomarkers (NfL), as detailed in later sections.
2. Acute neuroinflammatory response serves as the key secondary driver that extends beyond the initial transient metabolic crisis. In the developing pediatric brain, microglial priming and astrocytic activation alter ongoing myelination, synaptic pruning, and blood–brain barrier integrity, offering a direct mechanism for PPCS.

Together, these two interconnected pathways bridge hyperacute intracellular events with subacute clinical symptom profiling, diagnostic fluid biomarkers, and domain-targeted rehabilitation strategies.

### 4.1. Diffuse Axonal Injury

In addition to acute cellular and neurometabolic alterations, concussion involves early microstructural strain and functional disruption of white matter tracts connecting cortical and subcortical networks. Biomechanical shear and tensile forces acutely deform axons, impairing rapid signal transmission across neural pathways integral to higher-order cortical function. When widespread, this dynamic structural damage culminates in DAI, the classic pathological hallmark following diffuse TBI [38]. From a gross perspective, cortical coup–contrecoup injuries are implicated as part of the acute disruption of pathways in concussion [39]. Rather than representing a static, immediate mechanical tearing of nerve fibers, DAI involves a continuum of immediate mechanical strain and progressive secondary neurodegeneration. While extreme biomechanical forces can cause immediate primary axotomy, concussive impacts predominantly trigger secondary axotomy, a delayed, evolving process unfolding over hours to days post-injury. Biomechanical shear and tensile forces induce sub-lethal axolemmal mechanoporation, triggering pathological intra-axonal calcium influx. Elevated intracellular calcium persistently activates calpain and calcineurin proteases, which progressively degrade the spectrin sub-axolemmal cytoskeleton, neurofilaments, and microtubular tracks [40,41,42]. This progressive structural degradation impairs fast axonal transport, causing organelle accumulation and focal axolemmal swellings (axonal varicosities and retraction bulbs) [43]. Over time, localized transport blockage culminates in secondary axonal disconnection, Wallerian degeneration, and network-wide loss of structural connectivity [44].

Routine computed tomography (CT) or MRI is frequently negative in the majority of patients who sustain concussion and mTBI, mainly due to the microscopic nature of pathological changes [45,46,47]. Beyond acute cellular perturbations, imaging studies suggest that concussion and repetitive sub-concussive head impacts disrupt white matter connectivity, potentially affecting ongoing brain maturation in children and adolescents [48]. In recent years, DTI has been shown to measure alterations of white matter integrity following concussion, which have been interpreted as evidence for DAI [49,50,51].

Taken together, current evidence indicates that DAI should be viewed as a dynamic process of progressive microstructural disruption rather than an immediate mechanical event. This evolving axonal pathology likely contributes to persistent cognitive dysfunction, prolonged symptom recovery, and increased vulnerability to subsequent injury. Importantly, progressive axonal degradation, cytoskeletal breakdown, and cellular strain release damage-associated molecular signals that directly activate surrounding glial cells, providing a key physiological link to the acute neuroinflammatory response.

### 4.2. Acute Neuroinflammatory Response

Crucial to the evolving pathophysiological model of pediatric concussion is the acute neuroinflammatory response triggered by mechanical forces. Mechanical deformation of neural tissue prompts the rapid activation and morphological transformation of resident microglia and astrocytes [11]. Microglial priming leads to the acute release of pro-inflammatory cytokines, including interleukin-1 beta, interleukin-6, and tumor necrosis factor-alpha, which exacerbate synaptic dysfunction and local metabolic distress. Simultaneously, astrocytic reactivation and matrix metalloproteinase-9 upregulation drive transient breakdown of blood–brain barrier endothelial tight junction proteins (such as claudin-5 and occludin), permitting transient extravasation of serum proteins and systemic immune mediators into the brain parenchyma [11,52,53]. From a pediatric neurodevelopmental perspective, this neuroinflammatory response is uniquely distinct from that observed in the mature adult brain. In the developing central nervous system, resident microglia are already in an active, highly dynamic physiological state to support ongoing synaptogenesis, dendritic arborization, and activity-dependent synaptic pruning. Following mechanical insult, these developmentally active microglia exhibit heightened reactivity and an exaggerated pro-inflammatory response compared to adult microglia. Concurrently, because the pediatric neurovascular unit is still undergoing structural maturation, mechanical strain triggers more pronounced or prolonged blood–brain barrier (BBB) permeability alterations. Crucially, post-traumatic neuroinflammation directly interferes with key pediatric neurodevelopmental processes: elevated pro-inflammatory cytokines impair oligodendrocyte progenitor cell differentiation, disrupt active myelination of developing white matter tracts, and aberrantly alter synaptic pruning in prefrontal and limbic networks. This heightened developmental vulnerability provides a direct neurobiological mechanism explaining why identical biomechanical forces often lead to more protracted cognitive, affective, and somatic post-concussive recovery trajectories in children and adolescents compared to adults [7,11,27,54,55].

Emerging evidence therefore supports a multidimensional model of concussion in which neurometabolic dysfunction, DAI, CBF disturbances, and neuroinflammatory responses evolve simultaneously and interact throughout recovery. These overlapping mechanisms explain why clinical symptoms often persist despite apparently normal conventional neuroimaging findings.

### 4.3. Sex-Specific Differences in Pathophysiology, Symptomatology, and Recovery Trajectories

Biological sex significantly influences concussion susceptibility, biomechanical vulnerability, symptom presentation, and recovery trajectories in pediatric and adolescent populations. From a biomechanical perspective, adolescent females exhibit lower cervical muscle mass, smaller neck girth, and lower isometric neck strength compared to age-matched males, resulting in greater head peak angular acceleration and higher relative mechanical strain during equivalent impact forces. Mechanistically, sexual dimorphism in neuroinflammatory pathways, microvascular regulation, and hormonal fluctuations modulates post-injury blood–brain barrier permeability, neurovascular coupling, and microglial reactivity. Clinically, female youth consistently report a higher total symptom burden, elevated rates of somatic and affective dysfunction, and a significantly higher incidence of PPCS relative to male peers. Furthermore, preliminary biomarker studies suggest baseline sex-dependent variations in circulating brain-specific proteins. Integrating sex-specific considerations is therefore crucial for accurate risk stratification, clinical domain profiling, and personalized active recovery prescriptions [56,57,58,59,60,61,62,63].

In summary, pediatric concussion pathophysiology represents a dynamic, multi-tiered process initiated by hyperacute ionic and neurometabolic disturbances, which sequentially evolve into progressive microstructural DAI and a secondary neuroinflammatory response. In the developing central nervous system, these cellular mechanisms are uniquely magnified by neurodevelopmental vulnerabilities and modulated by sex-specific biomechanical and hormonal factors. Recognizing this complex pathophysiological continuum provides the neurobiological foundation for replacing legacy numerical severity grading with domain-based clinical profiling and early active rehabilitation strategies.

## 5. Symptomatology

Historically, concussion symptoms have been broadly grouped into four traditional categories: physical/somatic, cognitive, emotional/affective, and sleep disturbances [3,64]. However, to align with modern consensus frameworks (Amsterdam 2022/2023; Table 1), clinical evaluation now categorizes these manifestations into five distinct, actionable clinical domains: (1) somatic; (2) cognitive; (3) vestibular-ocular; (4) affective/emotional; and (5) sleep-related [7,8]. Distinguishing vestibular-ocular dysfunction as an independent domain represents a critical refinement over legacy four-category systems, as it directly guides targeted neuro-rehabilitative therapies (such as VOMS screening and specialized physical therapy).

Physical and cognitive symptoms are reported as the most common, while sleep disturbances and emotional symptoms are reported as the most bothersome [3,64].

Despite the presence of significant symptomatology and acute disability, routine neuroimaging (CT or standard MRI) is typically unhelpful at demonstrating evidence of neuropathologic changes. These symptoms are likely caused by functional, metabolic, and microstructural abnormalities, including ionic imbalance, metabolic dysfunction, and DAI, as opposed to gross macrostructural damage [10,27,47].

In general adult populations, clinical symptom resolution typically follows a rapid sequential course, with approximately 80–90% of concussed adults becoming symptom-free within 7 to 10 days post-injury [65]. In contrast, pediatric and adolescent populations exhibit a significantly prolonged recovery trajectory; up to 30% of concussed children remain symptomatic at one month post-injury due to unique neurodevelopmental vulnerabilities [27,64].

Across both pediatric and adult populations, post-concussive symptoms encompass this broad multidomain spectrum, spanning somatic, cognitive, vestibular-ocular, affective, and sleep-related domains [65,66,67,68,69,70,71,72,73,74]. While symptoms dissipate within 10–30 days for the majority of individuals [74,75,76], epidemiologic data from general mTBI cohorts indicate that up to 23% of patients experience persistent symptoms extending up to one year post-injury [77], with long-term cognitive deficits occurring predominantly in those with a history of repetitive head impacts [78]. In pediatric cohorts specifically, PPCS is clinically defined by symptom persistence beyond 4 weeks post-injury, affecting approximately 15–30% of youth [22,79].

Overall, the broad spectrum of clinical manifestations reflects the heterogeneous biological mechanisms underlying concussion. Consequently, contemporary management increasingly emphasizes symptom-domain identification rather than assuming a uniform clinical presentation or recovery trajectory.

## 6. Chronic Changes and Consequences of Concussion

While the majority of pediatric patients achieve clinical recovery within 2 to 4 weeks following mild traumatic brain injury, a subset exhibits prolonged symptomatology, with approximately 15–30% experiencing PPCS extending beyond one month [22,79]. The development of PPCS is multifactorial, driven by an interaction of premorbid patient vulnerability, acute injury characteristics, and neurodevelopmental factors. Key prognostic risk factors for protracted recovery include older adolescent age, female sex (mediated by cervical biomechanical differences, hormonal influences on neuroinflammation, and higher reported acute symptom burden), pre-existing migraine or psychiatric history (such as anxiety or depression), prior concussion history, high acute symptom burden, and initial post-traumatic amnesia [80,81,82]. To operationalize these risk factors in acute care settings, validated pediatric emergency risk stratification tools (such as the 5P score) integrate acute clinical parameters to identify high-risk children who require early, targeted follow-up [79].

Beyond acute physical symptoms, chronic sequelae of pediatric concussion encompass persistent neurocognitive, academic, and affective impairments. Long-term neurocognitive deficits commonly manifest as reduced processing speed, working memory dysfunction, and executive impairment, which directly impact academic performance and social reintegration [12,78]. Furthermore, persistent affective disturbances, including secondary depression and elevated anxiety, are frequently reported in pediatric cohorts with protracted trajectories [67,75]. While long-term neurodegenerative conditions remain primarily characterized in adult contact-sport and military populations, the cumulative burden of repetitive concussive and sub-concussive impacts during critical pediatric neurodevelopmental windows underscores the need for vigilant long-term tracking [83,84]. Establishing these chronic clinical outcomes provides the biological and functional rationale for systematic evaluation (Section 7) and domain-targeted management protocols (Section 10).

## 7. Evaluation of Injury and Neurophysiological Assessment

Whether a concussion has occurred is defined by the following symptoms: alterations in cognitive functioning (confusion/disorientation, amnesia), significant noncognitive signs (imbalance, vertigo, ringing/buzzing in the ears, sleep disturbances, fatigue, headache), or mood changes (anxiety, depression, agitation) [7,8]. The GCS is the standard for assessing the level of consciousness on a 3–15 rating scale, with scores of 13–15 defining mTBI [19].

For standardized acute and subacute clinical evaluation, the Concussion in Sport Group introduced updated, age-tailored diagnostic toolsets [7,8,9]:

- Child SCAT6: Standardized instrument designed for acute sideline and emergency department assessment of children aged 8 to 12 years (used within the first 72 h post-injury) [9].
- Child SCOAT6: Comprehensive, multidisciplinary assessment tool designed for clinical office environments for evaluations conducted beyond 72 h, integrating detailed neurocognitive, cervical, vestibular, and ocular-motor profiling [8].
- Vestibular/Ocular Motor Screening: Essential clinical screening tool that evaluates smooth pursuit, saccades, convergence (near point of convergence distance), vestibular-ocular reflex, and visual motion sensitivity. A positive Vestibular/Ocular Motor Screening is highly sensitive in identifying pediatric vestibular/oculomotor deficits and predicts protracted recovery [85,86].

Early identification of children at risk for protracted recovery remains critical in the acute setting. Clinical risk modeling in pediatric emergency departments allows clinicians to predict PPCS based on initial clinical presentation and domain-specific symptom scores [79].

Only after initial acute safety and clinical screening tools (e.g., Child SCAT6/SCOAT6) have been completed [7,8,9], formal neuropsychological assessment may be considered as a complementary clinical evaluation. Rather than adhering to rigid, fixed post-injury testing schedules (e.g., mandatory testing at 24–48 h or day 5), contemporary consensus guidelines (e.g., Amsterdam 2022/2023 framework and Centers for Disease Control and Prevention guidelines) emphasize that comprehensive neuropsychological testing should be deployed selectively and flexibly based on clinical indication [1,7]. Primary indications include evaluating complex or protracted recovery trajectories, characterizing specific domain deficits, and guiding individualized return-to-learn academic accommodations [1,7,8,30]. When administered, serial testing intervals should be determined by clinical recovery progression rather than arbitrary chronological timelines, thereby minimizing patient burden, acute fatigue, and practice-effect artifacts [7,30]. The general experience is that single uncomplicated mTBIs usually result in transient neuropsychological impairment with rapid resolution [30,74]. Symptoms may persist for extended periods of time, however, even after the resolution of cognitive changes, and there may also be cumulative effects of cerebral concussions.

While neuropsychological and neurophysiological assessments offer valuable insights into functional recovery, their clinical application must be interpreted within the context of key diagnostic limitations and operational challenges.

First, a direct correlation between patient-reported symptoms and objective test performance is not universally present; clinical symptom–cognitive discordance is frequently observed, wherein subjective symptoms (e.g., headache, fatigue, anxiety) persist long after cognitive scores have normalized, or conversely, objective cognitive deficits remain undetected by subjective symptom scales [30]. Second, the diagnostic accuracy and test–retest reliability of serial testing are heavily influenced by developmental variability, baseline intellectual functioning, test–retest practice effects, submaximal effort, and state-dependent factors such as fatigue, pain, and mood lability. Third, in monitoring disease progression, brief cognitive screening batteries often exhibit ceiling effects that may fail to capture subtle executive dysfunction or processing speed deficits under high academic or physical exertion.

Consequently, neuropsychological testing should not be utilized as a standalone diagnostic arbiter or sole clearance criterion for RTS. Rather, its primary role in contemporary clinical management is to characterize specific functional deficits, inform tailored RTL academic accommodations, track protracted recovery trajectories in complex cases, and guide targeted multidisciplinary intervention [7,8,30].

## 8. Neuroimaging Diagnostics

Clinical neuroimaging studies are not routinely acquired for patients who sustain concussion unless there is concern for intracranial injury that may necessitate intervention. Clinical CT and MRI findings are typically negative on the occasions when neuroimaging is utilized in the acute/subacute phase of concussion [87,88,89].

DTI is an advanced magnetic resonance technique that maps the microstructural integrity and spatial orientation of white matter tracts by measuring the directional movement (anisotropy) of water molecules along axonal fibers. In concussion and mTBI, where routine CT and standard structural MRI routinely fail to detect macroscopic lesions, DTI warrants particular attention because it provides a non-invasive in vivo probe for microstructural axonal strain and subclinical DAI. By quantifying key directional metrics (such as fractional anisotropy, which reflects axonal alignment and membrane integrity, and mean diffusivity, which indicates overall cellular density and edema), DTI can detect subtle tract disruptions (e.g., in thalamo-prefrontal and corpus callosum pathways) that correlate directly with acute neurocognitive deficits and protracted symptom trajectories [12,49,90].

Regarding DTI, multiple factors may affect its results, including the timing of assessment post-injury, the examination of specific regions of interest, patient age, or methods used to analyze DTI data [90,91,92].

Interpretation of DTI findings remains challenging because published studies have reported inconsistent alterations in fractional anisotropy and diffusivity measures, partly reflecting differences in scanner hardware, acquisition protocols, post-processing methods, timing of image acquisition after injury, and selected regions of interest. Multisite studies have demonstrated robust scanner effects on DTI metrics, emphasizing the need for data harmonization to reduce false positives and improve reproducibility. Furthermore, the number of diffusion-encoding directions affects the accuracy and precision of DTI measures, with a minimum of 18 directions recommended for reliable results [93,94]. Consequently, DTI currently remains primarily a research tool, and further methodological standardization is required before widespread clinical implementation.

Routine clinical neuroimaging is not indicated for pediatric patients with uncomplicated concussion. In particular, head CT should be used judiciously to minimize unnecessary exposure to ionizing radiation. While MRI does not involve ionizing radiation, its acute clinical utility is limited by cost, availability, acquisition duration, and the potential need for sedation in younger pediatric patients [1,16]. Emergency evaluation should strictly adhere to validated decision rules such as the Pediatric Emergency Care Applied Research Network (PECARN) criteria to rule out clinically important TBI [16]. When concurrent cervical spine involvement is suspected due to midline neck tenderness, focal sensory/motor deficits, or severe neck pain, clinical decision rules such as the NEXUS criteria should guide the selective use of cervical radiography or MRI [95].

Overall, conventional neuroimaging remains indispensable for excluding clinically important structural injuries, whereas advanced imaging techniques provide valuable research insights into the microstructural consequences of concussion. Future clinical integration of advanced neuroimaging will require greater methodological standardization and validation.

### Clinical Recommendations for Neuroimaging in Pediatric Concussion

Based on current evidence and consensus guidelines, the following approach is recommended:

1. Emergency CT imaging should be guided strictly by validated decision rules, such as the PECARN criteria [16]. Immediate head CT is indicated for high-risk features (e.g., GCS < 15, altered mental status, or signs of palpable/basilar skull fracture). For children presenting with intermediate-risk features (e.g., isolated LOC, severe injury mechanism, worsening headache, or abnormal behavior according to caregivers), clinical observation for 4–6 h is a well-established and appropriate option prior to deciding on neuroimaging [16].
2. Standard MRI without contrast is not routinely indicated in the acute setting but may be considered for patients with persistent neurological deficits, worsening symptoms, or suspected intracranial pathology [1,7].
3. Advanced MRI techniques (DTI, functional MRI, magnetic resonance spectroscopy) should currently be reserved for research settings or specialized clinical centers until standardized protocols and normative pediatric data are established [47].
4. Cervical spine imaging should be guided by NEXUS criteria or similar validated decision rules when cervical injury is suspected [95].

In summary, while routine neuroimaging (CT and standard structural MRI) is unhelpful for detecting functional concussive injury, acute head CT remains indispensable for ruling out life-threatening neurosurgical emergencies, guided strictly by validated decision rules such as PECARN to minimize ionizing radiation exposure. Advanced MRI modalities, particularly diffusion tensor imaging, offer powerful in vivo insights into microstructural white matter strain and subclinical DAI, but require further acquisition and analytical standardization before routine clinical deployment can be established.

## 9. Fluid Biomarkers for Diagnostic Triage, Prognostication, and Longitudinal Disease Progression Monitoring

Over the past decade, blood-based fluid biomarkers have emerged as pivotal adjuncts in the acute diagnostic triage and risk stratification of pediatric mTBI. Mechanistically, structural damage to central nervous system cells releases brain-specific proteins into the interstitial fluid, which subsequently cross the compromised blood–brain barrier into the systemic circulation. Among these, GFAP, a monomeric intermediate filament protein expressed in the astrocytic cytoskeleton, and UCH-L1, a neuron-specific deubiquitinating enzyme, have demonstrated high diagnostic accuracy for detecting acute intracranial lesions in adult populations [96]. However, their diagnostic performance in pediatric mTBI remains under active investigation in ongoing prospective clinical trials [14]. These markers exhibit complementary temporal kinetics: UCH-L1 elevates rapidly within hours of injury, peaking at approximately 8 to 12 h post-impact and declining sharply over 24 to 48 h (serum half-life of ~10 h), whereas GFAP levels rise steadily and remain persistently elevated for up to seven days, providing a broader clinical window for evaluation [96,97,98].

While GFAP and UCH-L1 have received Food and Drug Administration clearance for acute intracranial lesion triage on CT in adult populations, their direct extrapolation to pediatric mTBI remains constrained by developmental variations in baseline circulating levels. Pediatric neurodevelopment, ongoing myelination, and BBB maturation result in age-dependent biomarker kinetics. Consequently, ongoing prospective pediatric cohort validations (such as the BRAINI-2 trial and pediatric emergency network registries) are actively defining normative, age-stratified reference brackets and validated clinical decision cut-offs to safely translate these point-of-care assays into pediatric emergency triage algorithms [14,96].

In acute emergency care, the primary clinical utility of fluid biomarkers lies in ruling out intracranial pathology to minimize unnecessary diagnostic radiation from head CT scans. Following Food and Drug Administration clearance of plasma GFAP and UCH-L1 assays for adult trauma triage, prospective pediatric validation trials have established age-adjusted pediatric reference intervals [14,96]. When integrated alongside established clinical decision rules such as PECARN, high-sensitivity GFAP/UCH-L1 assays offer strong potential to assist in ruling out intracranial injury; however, while near 100% negative predictive values have been demonstrated in adult cohorts, pediatric-specific diagnostic cut-offs are still undergoing prospective validation before routine emergency department implementation can be recommended [14,16]. Conversely, while S100 calcium-binding protein B (S100B) is integrated into European Scandinavian trauma guidelines, its diagnostic specificity in young children remains restricted due to cross-reactivity with extracranial osteoblastic activity during active skeletal maturation. A recent meta-analysis of pediatric TBI studies reported a pooled sensitivity of 98% but a specificity of only 45% for S100B, with high heterogeneity across studies. This limits its utility as a stand-alone diagnostic tool in pediatric populations, and further studies are needed to establish pediatric-specific cut-off levels [96,99].

Consequently, blood-based biomarkers establish a continuous diagnostic, prognostic, and longitudinal monitoring framework that bridges hyperacute emergency department triage with subacute outpatient management. Beyond acute CT triage and baseline risk stratification, serial biofluid sampling provides a critical objective window into disease progression, resolution of neuroinflammation, and structural neural recovery trajectories over time. While acute GFAP and UCH-L1 testing serves as an immediate decision-support tool to exclude CT-visible structural lesions, serial measurement of astrocytic (GFAP) and axonal markers (NfL, total tau) allows longitudinal tracking of evolving neurodegenerative versus reparative processes [33,96,100]. NfL and total tau exhibit delayed peak kinetics and prolonged systemic elevation, directly reflecting ongoing white matter tract degradation and DAI kinetics [33,100]. Serial monitoring of serum NfL and GFAP clearance curves offers exploratory utility for observing recovery trajectories beyond the acute window and detecting potential subclinical cumulative injury in youth with protracted recovery or repeated impacts [33,101,102]. However, while ongoing research investigates whether fluid biomarkers track underlying cellular recovery, current pediatric evidence remains insufficient to mandate biomarker normalization as a criterion for return-to-sport clearance, which must continue to rely on standardized clinical, functional, and exertional evaluation [1,7,101,102].

However, translating these diagnostic and prognostic biomarker frameworks into routine pediatric practice requires addressing key neurodevelopmental physiological caveats. Recent studies have characterized age-dependent differences in the blood levels of GFAP and UCH-L1 in children without TBI [15]. A significant negative association between age and GFAP levels has been demonstrated [13,103], suggesting that diagnostic cut-offs for TBI based on GFAP may need to be adjusted in children younger than 11 years [89]. In contrast, UCH-L1 levels do not show the same age-dependent variation [15,50,104], potentially making it a more stable biomarker across pediatric age groups. The CALIPER cohort study established three age partitions for GFAP: 1 to <3.5 years (80.4–601 pg/mL); 3.5 to <11 years (50.7–224 pg/mL); and 11 to <19 years (26.2–119 pg/mL) [13]. These findings underscore the critical clinical distinction between age-related normative reference intervals and clinically validated diagnostic cut-offs. Normative reference intervals (such as those established by the CALIPER cohort [13]) define baseline, non-injured biomarker concentration distributions across pediatric growth stages. In contrast, clinically validated diagnostic cut-offs represent specific operational thresholds calibrated to rule out CT-visible intracranial lesions or predict clinical outcome trajectories [14,15]. Because baseline GFAP concentrations naturally decline with advancing age during childhood, applying adult diagnostic thresholds to pediatric patients risks substantial misclassification. Establishing age-adjusted, clinically validated decision cut-offs, distinct from healthy reference ranges, remains an essential prerequisite for routine pediatric biomarker implementation [13,15,105].

Despite these promising developments, several important challenges remain before blood biomarkers become part of routine pediatric concussion care. Age-dependent physiological variability, differences between analytical platforms, uncertain diagnostic thresholds, and the limited number of large prospective pediatric validation studies currently restrict widespread implementation. Consequently, fluid biomarkers should presently be regarded as complementary diagnostic tools that augment, rather than replace, careful clinical evaluation and established decision rules.

The principal characteristics, clinical applications, and current limitations of the most extensively investigated fluid biomarkers in pediatric concussion are summarized in Table 2.

## 10. Patient Management

After a patient with a suspected concussion is identified, standard medical procedures should first be applied (ensuring an open airway and adequate circulation). Stabilizing the cervical spine should be conducted if injury is suspected. Serial monitoring to detect any acute deterioration in mental status is critical over the first few hours following injury, and the patient should not be left alone during this initial observation window [1,7,106]. However, once red-flag symptoms and acute structural complications have been clinically excluded, routine nighttime awakening is unnecessary and discouraged; normal, uninterrupted restorative sleep should be encouraged, as forced nocturnal sleep disruption can exacerbate post-concussive fatigue, anxiety, and overall symptom burden [1,7].

Understanding whether symptoms have improved, worsened, or remained the same since injury is also important. Combining information from the history and physical examination with an understanding of symptom severity allows determining what course of action should be taken [1,7].

The patient should not be left alone and should be transported to the emergency department if any of the following is present [1,7,106]:

- A headache that worsens,
- Drowsiness or inability to be woken up,
- Inability to recognize people or places,
- Repeated vomiting,
- Worsening confusion/irritability,
- Seizures,
- Hemiparesis/hemisensory loss,
- Unsteadiness, or
- Slurred speech.

Transportation to the emergency department by ambulance is recommended if there is suspicion of any of the following: spinal cord involvement, focal neurologic deficit, worsening of condition suggestive of intracranial hematoma, indicators of a skull fracture, bleeding from the nose or ear, or a seizure [1,7].

### 10.1. Practical Integration of the Multimodal Decision Framework in Clinical Practice

Operationalizing a multimodal framework requires a sequential, tier-based clinical workflow that categorizes diagnostic tools by clinical phase, diagnostic priority, and functional domain:

- Tier 1: Acute Structural Safety and Triage (0–72 Hours Post-Injury)

In acute emergency or sideline settings, the primary objective is excluding life-threatening macrostructural injury while avoiding unnecessary ionizing radiation. Clinical decision rules (PECARN) hold highest priority; high-risk features dictate immediate head CT [16]. In intermediate-risk cases, hyperacute fluid biomarkers (GFAP/UCH-L1) serve as adjunctive triage tools to reinforce decision-making against unnecessary imaging as age-adjusted cut-offs are validated [14,96]. Concurrently, the Child SCAT6 is deployed to establish acute symptom burden, baseline balance, and immediate cognitive function [9].

- Tier 2: Subacute Multidomain Profiling (>72 Hours Post-Injury)

In outpatient clinical settings, management shifts from structural triage to functional domain profiling. The Child SCOAT6 serves as the central diagnostic backbone, integrating detailed symptom checklists, medical history, and mental status testing [8]. Within this framework, specific diagnostic instruments are weighed according to clinical presentation:

o Vestibular-Ocular Phenotyping: The VOMS assessment is weighted heavily if patients report dizziness, motion sensitivity, or visual fatigue. Positive VOMS findings directly trigger referral for targeted vestibulo-ocular physical therapy [85,86].
o Cervical & Somatic Phenotyping: Cervical spine evaluation (NEXUS criteria) and postural stability testing dictate whether physical therapy or cervical rehabilitation is prioritized [95].
o Autonomic & Exertional Phenotyping: Treadmill/cycle exertion testing (e.g., Buffalo Concussion Treadmill Test) determines individual heart-rate thresholds for early sub-symptom aerobic exercise [17].

- Tier 3: Decision Integration and Recovery Tracking

Clinical decision-making in this framework relies on a hierarchical rule: academic reintegration takes clinical priority over athletic clearance. Domain profiles directly inform accommodations. For example, cognitive fatigue prompts RTL adjustments, whereas vestibulo-ocular deficits guide physical therapy alongside light aerobic activity. Progress is monitored longitudinally using serial SCOAT6 domain scores. In cases of PPCS (>4 weeks), longitudinal fluid biomarkers such as NfL may be weighed alongside advanced MRI (DTI) to detect ongoing subclinical microstructural axonal strain and guide multidisciplinary specialist referral [33,101].

### 10.2. Rest and Early Active Recovery/Sub-Symptom Aerobic Exercise

Management of pediatric concussion has fundamentally evolved from historical prolonged strict rest (‘dark room therapy’) or complete sensory deprivation (‘cognitive blackout’) [107,108,109]. Current consensus emphasizes relative rest during the initial 24 to 48 h post-injury [6,7]. Relative rest is defined as engaging in basic activities of daily living and reduced, non-provocative screen time/cognitive activity while avoiding strenuous physical exertion, rather than enforcing total bed rest [6,109]. Following this brief initial period of relative rest, pediatric patients should be encouraged to progressively increase daily activity and initiate sub-symptom threshold aerobic exercise (e.g., stationary cycling, brisk walking) [6,17].

Nevertheless, important questions remain regarding the optimal timing, intensity, duration, and progression of aerobic exercise following pediatric concussion. Current recommendations are primarily based on symptom-limited individualized protocols, and additional randomized studies are needed to determine the most effective exercise prescription for different patient subgroups.

### 10.3. Return-to-Learn and Return-to-Sport Progression

For pediatric and adolescent patients, RTL and RTS are complementary, interdependent recovery pathways that should proceed in parallel, with academic reintegration taking clinical priority over athletic competition [1,7]. Early stages of RTS (e.g., symptom-free daily activities, light aerobic exercise, and individual non-contact sport-specific drills; Stages 1–3) can occur concurrently while a student is actively receiving RTL classroom accommodations (e.g., temporary half-days, reduced screen time, frequent rest breaks, and deferred testing) [7,8]. However, full academic recovery (defined as full-time school attendance with complete workload tolerance and no required academic accommodations) is a mandatory prerequisite before an athlete may advance to full-contact practice, risk-bearing training drills, or unrestricted competitive sport (RTS Stages 5–6) [1,7]. In accordance with international consensus, same-day return to sport following a suspected or confirmed concussion is strictly contraindicated in all pediatric and adolescent athletes [1,7].

### 10.4. Modern Diagnostic Staging and Severity Classification

Historically, acute concussion management relied on rigid numerical grading systems based primarily on LOC and post-traumatic amnesia. However, these legacy frameworks have been fully retired because acute severity grades fail to predict clinical trajectory, prolonged recovery, or secondary risk in pediatric populations [1,7]. Under contemporary consensus guidelines (Amsterdam 2023; Centers for Disease Control and Prevention), concussion is classified at the mild end of the acute TBI spectrum (Table 3), requiring individualized symptom-driven risk stratification and multidomain clinical profiling rather than fixed numerical grading [1,7,21].

Taken together, current evidence demonstrates that successful concussion management extends beyond symptom resolution and requires individualized progression through cognitive, physical, and sport-specific rehabilitation. Early active recovery combined with structured RTL and RTS strategies currently represents the cornerstone of modern pediatric concussion care. The principal conceptual changes that have transformed pediatric concussion diagnosis and management over the past two decades are summarized in Table 4.

## 11. Second-Impact Syndrome and Repeated Injury

Minor head trauma may cause pathological alterations in potassium and calcium levels, can produce a physiological mismatch between oxygen/glucose demand and delivery, and can cause a loss of cerebral autoregulation, all of which may lead to increased vulnerability. Second-impact injuries typically present with signs and symptoms of a post-concussion syndrome (visual, motor, or sensory abnormalities and difficulties with cognitive processes).

If a second head impact occurs during this vulnerable metabolic window (before full neurofunctional recovery) it can trigger catastrophic loss of cerebral blood flow autoregulation, rapid vascular engorgement, and malignant diffuse cerebral edema [110,111,112,113,114,115]. True second-impact syndrome (SIS) is an exceptionally rare phenomenon, with fewer than 60 confirmed cases documented in the medical literature, almost exclusively involving children, adolescents, and young adults under 20 years of age due to age-specific cerebrovascular vulnerabilities [110,111,112,113,114,115]. Although its overall population incidence is extremely low, confirmed SIS carries a high mortality rate approaching 50% and near-universal severe neurological morbidity [113,114,116,117,118].

Beyond rare catastrophic events such as SIS, recurrent concussive and sub-concussive impacts more commonly alter recovery trajectories and lead to cumulative neurological burden. The time required to achieve complete clinical and neurocognitive recovery is closely related to initial injury severity and post-traumatic amnesia duration, typically resolving within 10 to 30 days in uncomplicated pediatric concussions [27,74,83]. However, repeated injuries significantly prolong recovery duration, increase the risk of PPCS, and exacerbate underlying white matter microstructural strain [82,83,84].

Additionally, patients with repeated concussion also appear to have a longer duration of symptoms [83]. A significant clinical dilemma arises in patients with repetitive concussions who report persistent subjective or functional deficits despite completely normal results on routine clinical testing modalities (such as conventional CT, standard structural MRI, and basic bedside screening tools). In these cases, subtle microstructural axonal disruption, neurometabolic dysregulation, or persistent neuroinflammation may evade standard clinical detection, underscoring the clinical utility of advanced modalities (e.g., diffusion tensor imaging, functional MRI, and blood fluid biomarkers) to identify subclinical or ongoing neural strain.

In patients aged less than 21 years, trauma-related injuries account for 14% of deaths, while the estimated rate of death from trauma is 0.11/100,000 participants, with an average of nine deaths per year [118]. Case reports exist of patients who suffer an initial mTBI, and after return to play and a second minor blow, quickly deteriorate and die. In many of these cases, an acute space-occupying mass, such as a hematoma, was not detected, and death was thought to result from severe and rapid brain edema [114,116,117,118]. It is known that in severe TBI, loss of autoregulation in association with rapid catecholamine release can lead to severe brain edema [115]. Subdural hematoma and edema are likely secondary to shear forces and SIS [114].

While true SIS, characterized by catastrophic cerebral edema following a second impact during the acute recovery phase, remains extremely rare, with fewer than 60 confirmed cases documented in the literature [110,111,112,113,114,115], the neurodevelopmental risks of repetitive head impacts in youth are well-established [1,7]. Repetitive concussive and sub-concussive impacts sustained during vulnerable neurodevelopmental windows can result in cumulative neurocognitive deficits, PPCS, and protracted clinical recovery [7,10]. The biological mechanism involves the post-injury period of cerebral vulnerability when cells are metabolically compromised and more susceptible to injury [26]. Rather than focusing exclusively on the rare catastrophic SIS, contemporary concussion management emphasizes prevention through [1,21,88]:

1. Strict adherence to graduated RTS protocols;
2. Age-appropriate rules limiting contact in youth sports;
3. Comprehensive preseason concussion education;
4. Early identification and management of concussion symptoms.

In summary, while catastrophic second-impact syndrome remains an exceptionally rare clinical entity, the cumulative risk of repetitive concussive and sub-concussive impacts poses a significant threat to the maturing pediatric brain. Sustaining subsequent impacts during vulnerable neurometabolic windows prolongs recovery trajectories, exacerbates microstructural white matter strain, and increases the risk of PPCS. Consequently, strict adherence to graduated return-to-sport protocols, age-appropriate contact restrictions, comprehensive educational initiatives, and vigilant longitudinal monitoring remains paramount to safeguard long-term neurological health in youth.

## 12. Limitations

This review has several limitations. First, while predefined inclusion and exclusion criteria and independent dual-author screening were utilized in alignment with the Scale for the Assessment of Narrative Review Articles guidelines, this manuscript remains a narrative review rather than a registered systematic review or meta-analysis, which inherently introduces the possibility of selection bias. Second, the rapidly evolving nature of concussion research means that some emerging evidence, particularly regarding fluid biomarkers and advanced neuroimaging, may have been published after the literature search period. Third, the review primarily emphasized English-language publications, potentially excluding relevant studies published in other languages. Fourth, many recommendations, particularly those regarding biomarker thresholds and exercise prescription, are based on adult studies or limited pediatric data and require further prospective validation. Fifth, the included studies exhibit considerable heterogeneity in terms of study design, population characteristics, outcome measures, and diagnostic criteria for concussion, which limits the ability to draw direct comparisons or pool results quantitatively. Sixth, while basic pathophysiological mechanisms apply across all pediatric mTBI etiologies, much of the contemporary evidence base for active recovery, specialized diagnostic tools (e.g., Child SCAT6/SCOAT6), and stepped return protocols originates from sports-related concussion cohorts; caution is therefore warranted when extrapolating these specific management algorithms to non-sports mechanisms (e.g., motor vehicle accidents, falls, or non-accidental trauma) where co-occurring systemic injuries or multi-organ trauma may alter the recovery trajectories. Seventh, formal quality assessment of included studies (e.g., using tools such as QUADAS-2 or Newcastle–Ottawa Scale) was not performed, which may affect the interpretation of the evidence base. Despite these limitations, the review integrates the most current international consensus guidelines with contemporary evidence to provide a clinically oriented overview for practitioners managing pediatric concussion.

## 13. Conclusions and Future Directions

Pediatric concussion represents a major public health challenge within the >20 million annual global TBI cases, with up to 30% of affected children experiencing PPCS beyond 4 weeks. Clinical assessment has transitioned from obsolete numerical grading scales to domain-specific symptom profiling for mild TBI (GCS 13–15). Diagnostically, integrating clinical decision rules (PECARN) with emerging fluid biomarkers (GFAP/UCH-L1) holds promise for optimizing CT triage, though pediatric-specific diagnostic cut-offs require rigorous multicenter validation prior to widespread clinical adoption. Clinically and scientifically, replacing historical prolonged rest with brief relative rest (24–48 h) followed by sub-symptom threshold exercise and structured RTL protocols significantly accelerates neurofunctional recovery and reduces long-term morbidity, while same-day return to play remains strictly prohibited to protect the developing brain.

Despite significant consensus-driven advancements in pediatric concussion management, several core scientific and clinical evidence gaps remain unaddressed across the literature:

- *Neurometabolic-to-Phenotype Mapping:* A substantial gap persists in directly linking underlying acute cellular and microstructural disturbances (e.g., axonal shear, microglial priming, and white matter tract disruption identified on advanced DTI) to specific clinical symptom domains (somatic, cognitive, vestibular-ocular, affective).
- *Pediatric Biomarker Calibration:* Current fluid biomarker cut-offs (GFAP, UCH-L1, NfL) are largely adapted from adult TBI cohorts. Developmentally driven baseline fluctuations—influenced by ongoing myelination, blood–brain barrier maturation, and active skeletal growth—hinder the immediate clinical implementation of standardized point-of-care triage assays in younger pediatric populations.
- *Granular Exercise Prescription Rules:* While early sub-symptom threshold exercise has proven beneficial, exact dose–response parameters—specifically the precise optimal heart rate threshold, daily duration, and subgroup-specific start times (e.g., <12 years vs. older adolescents)—remain inadequately defined.
- *Prospective Longitudinal Tracking of Cumulative Strain:* High-quality prospective data tracking the long-term neurodevelopmental, cognitive, and psychiatric trajectories of children exposed to repetitive concussive and sub-concussive head impacts into early adulthood remain limited.

To bridge these gaps and advance pediatric concussion care, future research initiatives should prioritize:

- *Longitudinal Biomarker Tracking for Disease Progression:* Investigating serial biofluid dynamics (GFAP, NfL, total tau) across subacute recovery phases to establish normative biological clearance curves, evaluate objective responses to active rehabilitation, and track longitudinal disease trajectories in pediatric cohorts.
- *Age- and Sex-Stratified Biomarker Calibration:* Establishing rigorous, prospective, age- and sex-stratified reference intervals and clinical decision cut-offs for circulating GFAP, UCH-L1, and NfL across pediatric developmental stages to account for sex-specific physiological baselines.
- *Multimodal Diagnostic and Prognostic Algorithms:* Validating integrated machine-learning and risk-stratification models that combine clinical domain profiling (Child SCOAT6, VOMS), fluid biomarkers, and advanced neuroimaging (DTI) to accurately predict PPCS in emergency and outpatient settings.
- *Domain-Targeted Rehabilitation Trials:* Conducting randomized controlled trials evaluating subgroup-specific, domain-targeted therapies (e.g., targeted vestibulo-ocular physical therapy versus sub-threshold aerobic protocols) to determine optimal individualized treatment algorithms.
- *Implementation Science and Global Translation:* Developing and evaluating scalable clinical decision-support systems to effectively translate international consensus guidelines (Amsterdam 2022/2023 framework) into primary care, school health systems, and resource-limited emergency departments.

Implementing a practical, tier-based clinical workflow that includes sequencing acute structural triage (PECARN, acute biomarkers), followed by subacute multidomain profiling (Child SCOAT6, VOMS) and targeted active rehabilitation, provides an actionable roadmap for translating multimodal concussion strategies into routine practice.

## Figures and Tables

**Table 1:** Clinical domain profiling in pediatric concussion (Amsterdam 2022/2023 framework) [7].

Clinical Domain	Primary Symptoms & Signs	Targeted Assessment & Intervention
Somatic	Headache, neck pain, nausea, light/noise sensitivity	Symptomatic analgesia, cervical spine physiotherapy, light sub-threshold activity
Cognitive	Processing speed delay, memory deficit, mental fatigue	Return-to-Learn accommodations, reduced screen time, cognitive rest
Vestibular-Ocular	Dizziness, vertigo, blurred vision, motion sensitivity, convergence insufficiency	Vestibular/Ocular Motor Screening (VOMS), targeted vestibulo-ocular physical therapy, vision therapy
Affective/Emotional	Irritability, anxiety, sadness, emotional lability	Psychological support, cognitive-behavioral strategies, parental education
Sleep-Related	Hypersomnia, insomnia, sleep-wake cycle disruption	Sleep hygiene protocol, blue-light exposure reduction, avoidance of late-day sedatives

**Table 2:** Most extensively investigated fluid biomarkers for pediatric concussion: current clinical applications and limitations.

Biomarker	Cellular Source	Principal Clinical Application	Advantages	Current Limitations
GFAP	Astrocytes	Identification of intracranial injury; acute CT triage; serial tracking of astrocytic injury resolution	High sensitivity for intracranial lesions; remains elevated for several days	Age-dependent reference ranges; limited pediatric validation; assay availability
UCH-L1	Neurons	Early detection of neuronal injury; CT triage	Rapid increase after injury; useful in hyperacute phase	Short diagnostic window; variability between assays; limited pediatric evidence
Neurofilament light chain	Axons	Assessment of diffuse axonal injury; prediction of prolonged recovery; investigational tracking of subacute white matter recovery trajectories	Reflects axonal damage; prognostic potential	Delayed peak concentration; uncertain clinical thresholds; not routinely available
Total tau	Axons	Evaluation of neuronal injury and recovery	May correlate with injury severity and persistent symptoms	Variable study results; low specificity; limited pediatric standardization
S100B	Astrocytes	Exclusion of intracranial injury (mainly European practice)	High negative predictive value in selected populations	Elevated after extracranial injuries; lower specificity in children due to skeletal growth

Blood biomarkers should currently be considered complementary diagnostic tools and interpreted alongside clinical assessment and validated decision rules; they are not validated clinical criteria for confirming biological recovery or determining return-to-sport clearance. Legend: GFAP—glial fibrillary acidic protein; UCH-L1—ubiquitin C-terminal hydrolase L1.

**Table 3:** Modern diagnostic and severity classification criteria for traumatic brain injury.

Clinical Parameter	Mild TBI (Concussion)	Moderate TBI	Severe TBI
Glasgow Coma Scale	13–15 (evaluated at 30 min post-injury)	9–12	3–8
Loss of Consciousness	<30 min (or none)	30 min to 24 h	>24 h
Post-Traumatic Amnesia	<24 h	1–7 days	>7 days
Structural Neuroimaging (CT/MRI)	Normal (uncomplicated) or non-operable lesion (complicated)	Normal or Abnormal	Abnormal
Numerical Grading Systems	Obsolete (Grades I–III no longer used)	N/A	N/A
Same-Day Return-to-Sport	Strictly Prohibited (Pediatrics)	Prohibited	Prohibited

Legend: TBI—traumatic brain injury; CT—computed tomography; MRI—magnetic resonance imaging.

**Table 4:** Evolution of pediatric concussion concepts and clinical management: historical versus contemporary evidence-based practice.

Aspect	Historical Approach	Contemporary Evidence-Based Approach
Concept of concussion	Primarily viewed as a transient injury caused by mechanical trauma	Dynamic neurometabolic, microstructural, vascular, and inflammatory process
Severity assessment	Numerical grading systems (Grades I–III) based mainly on loss of consciousness and symptom duration	Individualized multidomain clinical assessment with symptom-domain profiling
Role of loss of consciousness	Considered a major indicator of injury severity	Recognized as only one clinical feature with limited prognostic value
Neuroimaging	CT frequently obtained to exclude intracranial injury	Selective imaging guided by validated clinical decision rules (e.g., PECARN); advanced MRI techniques mainly research tools
Fluid biomarkers	Not available	Emerging adjunctive diagnostic and prognostic tools (GFAP, UCH-L1, NfL, t-Tau), complementing clinical assessment
Initial management	Prolonged strict physical and cognitive rest (“dark-room therapy”)	Brief relative rest (24–48 h) followed by individualized active rehabilitation
Exercise after concussion	Avoided until complete symptom resolution	Early sub-symptom threshold aerobic exercise recommended
Return-to-Learn	Rarely incorporated into management	Structured, individualized return-to-learn integrated into routine care
Return-to-Sport	Decisions based largely on symptom duration and historical grading scales	Stepwise progression after successful return-to-learn and complete clinical recovery
Overall management philosophy	Uniform recommendations for most patients	Personalized multidisciplinary management based on clinical domains, patient characteristics, and recovery trajectory

Contemporary recommendations are based primarily on recent international consensus statements and evidence-based clinical practice guidelines. Legend: PECARN—Pediatric Emergency Care Applied Research Network; CT—computed tomography; MRI—magnetic resonance imaging; GFAP—glial fibrillary acidic protein; UCH-L1—ubiquitin C-terminal hydrolase L1; NfL—neurofilament light chain; t-Tau—total tau.

## Data Availability

No new data were created or analyzed in this study. Data sharing is not applicable to this article.

## Abbreviations

The following abbreviations are used in this manuscript:

AP	Action potential
ATP	Adenosine-triphosphate
BBB	Blood–brain barrier
CBF	Cerebral blood flow
CT	Computed tomography
DAI	Diffuse axonal injury
DTI	Diffusion tensor imaging
EC	Extracellular
GCS	Glasgow Coma Scale
GFAP	Glial fibrillary acidic protein
Glu	Glutamate
IC	Intracellular
LOC	Loss of consciousness
MRI	Magnetic resonance imaging
mTBI	Mild traumatic brain injury
NfL	Neurofilament light chain
PECARN	Pediatric Emergency Care Applied Research Network
PPCS	Persistent post-concussive symptoms
ROS	Reactive oxygen species
RTL	Return-to-learn
RTS	Return-to-sport
SCAT6	Sport Concussion Assessment Tool 6
SCOAT6	Sport Concussion Office Assessment Tool 6
SIS	Second-impact syndrome
SRC	Sports-related concussion
TBI	Traumatic brain injury
t-Tau	Total tau
UCH-L1	Ubiquitin C-terminal hydrolase L1
VOMS	Vestibular/Ocular Motor Screening
